# Mechanistic Insights and Therapeutic Potential of the Antidepressant Amitriptyline against *Leishmania (Leishmania) amazonensis*

**DOI:** 10.1021/acsomega.5c04856

**Published:** 2025-08-07

**Authors:** Juliana Tonini Mesquita, Noemi Nosomi Taniwaki, Andre Gustavo Tempone, Juliana Quero Reimão

**Affiliations:** † Pathophysiology Laboratory, 196591 Instituto Butantan, São Paulo 01246-902, SP, Brazil; ‡ Electron Microscopy Nucleus, 89119 Instituto Adolfo Lutz, São Paulo 01246-000, SP, Brazil; § Laboratory of Preclinical Assays and Research of Alternative Sources of Innovative Therapy for Toxoplasmosis and Other Sicknesses (PARASITTOS), 146840 Faculdade de Medicina de Jundiaí, Jundiaí 13202-550, SP, Brazil

## Abstract

Leishmaniasis remains a significant global health challenge, with limited therapeutic options and rising drug resistance. The repurposing of Food and Drug Administration approved antidepressants as amitriptyline, a widely used tricyclic drug, could offer a promising strategy for developing novel antileishmanial agents. This study investigates the in vitro activity of amitriptyline against *Leishmania (Leishmania) amazonensis* promastigotes and intracellular amastigotes and explores its ultrastructural effects and potential in combination therapy. Amitriptyline was effective against the clinically relevant form, the intracellular amastigotes of *L. (L.) amazonensis*, resulting in an EC_50_ value of 22 μM. Ultrastructural analyses revealed mitochondrial swelling following amitriptyline treatment, suggesting mitochondria as a key target. This structural damage, in the absence of observable plasma membrane disruption, supports the hypothesis that amitriptyline may specifically target intracellular organelles rather than initiating cell lysis through membrane destabilization. Additionally, amitriptyline induces mitochondrial membrane depolarization in *L. (L.) amazonensis*, disrupting parasite energy homeostasis. Combination assays with standard drugs amphotericin B and miltefosine demonstrated additive interactions, reinforcing the potential of amitriptyline as complementary therapy. This work highlights the antileishmanial activity of tricyclic compounds and underscores the potential for further repositioning studies, broadening the assessment of clinically used, structurally related compounds.

## Graphical abstract

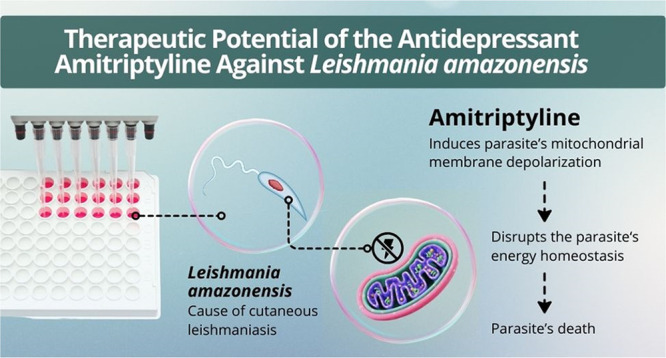

## 1 Introduction

Leishmaniasis is endemic in 99 countries and territories and remains a major public health concern in four eco-epidemiological regions: the Americas, East Africa, North Africa, and West and South Asia.^1^ Leishmaniasis, caused by intracellular protozoan parasites of the genus *Leishmania*, is classified as a Neglected Tropical Disease (NTD).^2^ More than 20 *Leishmania* species can infect humans, leading to clinical manifestations that range from cutaneous forms (the most common) to visceral leishmaniasis (the most severe and potentially fatal form).^2–5^

NTDs encompass 20 infectious diseases caused by bacteria, viruses, and parasites, disproportionately affecting impoverished populations in tropical and subtropical regions.^6,7^ Among them, leishmaniasis is responsible for high mortality rates and is further burdened by severe drug toxicity and emerging resistance.^7^ These limitations have intensified efforts to develop alternative therapies, with drug repurposing emerging as a key strategy. Repurposing involves identifying new therapeutic applications for approved drugs, accelerating drug discovery while reducing costs and bypassing early stage toxicity assessments.^4,7^

A promising approach in NTD research involves combining existing or repurposed drugs to enhance efficacy, prevent resistance, improve compliance, shorten treatment duration, and reduce overall costs.^7,8^ Tricyclic antidepressants have been shown antileishmanial activity and amitriptyline demonstrated activity against *Leishmania (Leishmania) donovani*,^9,10^ a causative agent of the visceral leishmaniasis in South Asia (India, Bangladesh, Nepal), Middle East and China.^11^ Amitriptyline is a tricyclic drug structurally related to the skeletal muscle relaxant cyclobenzaprine, differing only by the presence of a double bond in the central ring (Figure 1). Our previous studies demonstrated the in vitro and in vivo efficacy of cyclobenzaprine against *Leishmania (Leishmania) infantum*.^12^ Considering the structural similarity to cyclobenzaprine, amitriptyline was selected to be studied against *L. (L.) amazonensis*, a causative agent of the Brazilian cutaneous leishmaniasis (Figure 1). Amitriptyline is widely prescribed for depressive disorders and is also used off-label to treat conditions such as neuropathic pain, migraines, and anxiety.^13^ Building upon these findings, this study investigates the in vitro and ex vivo activity of amitriptyline against *L. (L.) amazonensis*, explores its potential mechanism of action, and assesses its effects in combination with clinically used antileishmanial drugs.

**Figure 1:**
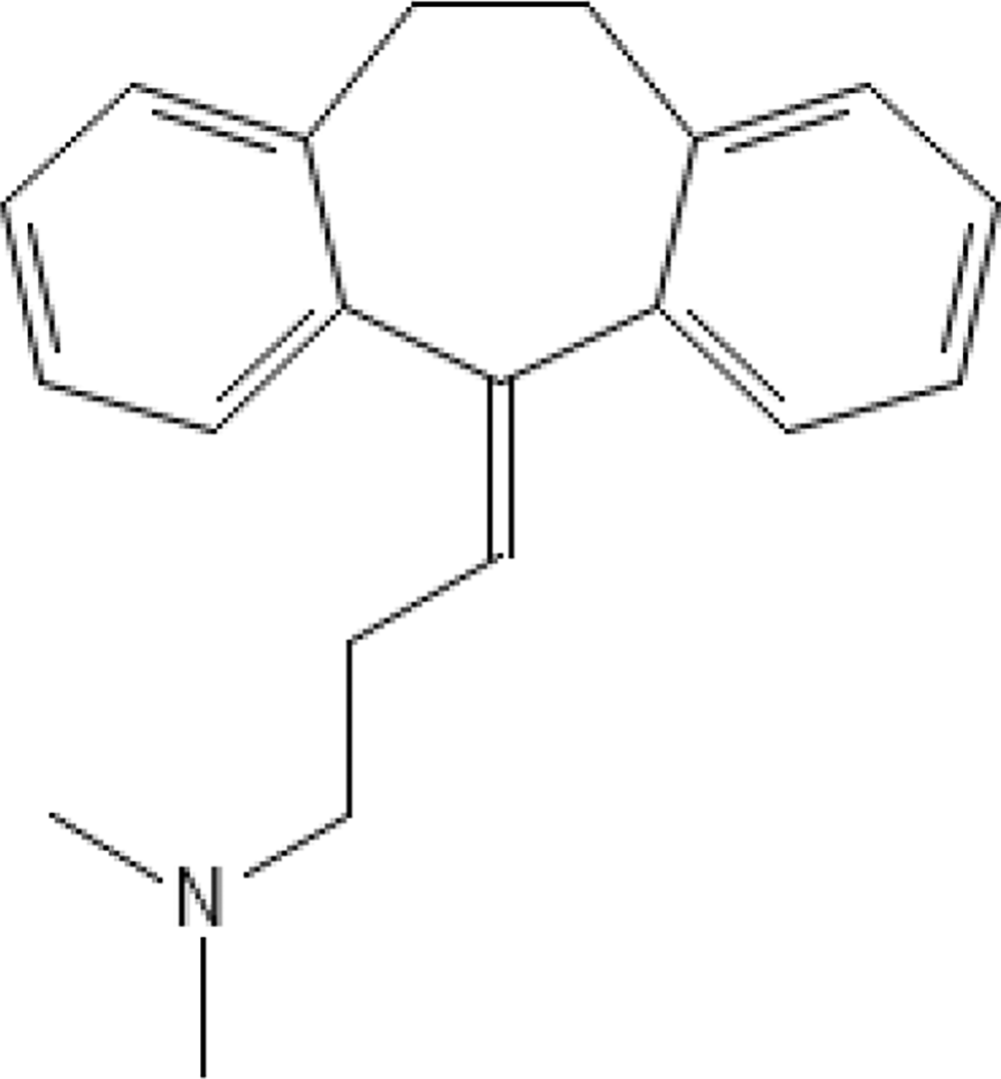
Chemical structure of the compound amitriptyline. Structure drawn by the authors using the PubChem Sketcher tool (https://pubchem.ncbi.nlm.nih.gov//edit3/index.html) from SMILE obtained in the PubChem library from the National Institutes of Health (NIH).^14^

## 2 Results

### 2.1 Antileishmanial Activity and Cytotoxicity against Mammalian Cells

The activity of the drug amitriptyline against promastigotes forms of *L. (L.) amazonensis* was determined using the 3-(4,5-dimethylthiazol-2-yl)-2,5-diphenyltetrazolium bromide (MTT) colorimetric method. Amphotericin B and miltefosine were used as standard drugs. After 48 h of incubation, amitriptyline resulted in 100% parasite mortality at the maximum tested concentration, with an Effective Concentration 50% (EC_50_) value of 8.4 μM and an Effective Concentration 90% (EC_90_) value of 44.8 μM. Amphotericin B and miltefosine showed EC_50_ values of 0.08 and 10.4 μM, respectively (Table 1).

**Table 1:** EC_50_, CC_50_, and SI Values of Amitriptyline and Reference Drugs against *Leishmania (Leishmania) amazonensis* and NCTC Cells^a^

drugs	EC_50_ and (μM) (±SD)		CC_50_ (μM) (±SD)	SI
	promastigotes	intracellular amastigotes	NCTC	
amitriptyline	8.4 ± 0.4	21.9 ± 5.8	142.9 ± 20.2	6.5
amphotericin B^b^	0.08 ± 0.02	0.02 ± 0.01	97.0 ± 7.2	4850.0
miltefosine^b^	10.4 ± 1.2	3.91 ± 1.5	179.6 ± 2.7	45.9

a EC_50_: effective concentration 50%; CC_50_: cytotoxic concentration 50%; SI: selectivity index (the ratio of the CC_50_ in NCTC cells and EC_50_ in intracellular amastigotes); SD: standard deviation.

b Standard drug in clinical use.

To determine the EC_50_ of the drugs against intracellular amastigote forms, parasite burden in infected macrophages was determined using a luminescent-based assay after 48 h of treatment. Amphotericin B and miltefosine were the most active, with EC_50_ values of 0.02 and 3.91 μM, respectively, while amitriptyline showed an EC_50_ value of 21.9 μM (Table 1) and an EC_90_ of 33.73 μM.

The cytotoxicity of the drugs against NCTC clone 929, a mouse fibroblast cell line, was determined after 48 h of treatment using the MTT method. Miltefosine had the highest cytotoxic concentration 50% (CC_50_) (indicating the lowest cytotoxicity), followed by amitriptyline and amphotericin B (Table 1).

Representative dose–response curves of amitriptyline, amphotericin B and miltefosine against *L. (L.) amazonensis* promastigotes and amastigotes and NCTC cells are shown in supplementary Figure 1.

Considering the ratio between CC_50_ in NCTC cells and EC_50_ in intracellular amastigotes, amphotericin B presented the highest Selectivity Index (SI), followed by miltefosine and amitriptyline (Table 1).

### 2.2 Mechanistic Insights

The assessment of plasma membrane integrity in promastigotes treated with amitriptyline was performed using the SYTOX Green probe, up to 60 min of drug incubation. This probe exhibits increased fluorescence when bound to DNA, indicating membrane rupture, pores, or alterations in parasite plasma membrane integrity. Maximum permeabilization in this experiment was achieved using the detergent Triton X-100, showing a significant alteration in plasma membrane integrity (***p < 0.0001) compared to untreated cells. Untreated promastigotes were used as controls to obtain baseline fluorescence. Readings taken every 10 min showed no increase in probe influx with amitriptyline treatment, indicating no alteration in plasma membrane integrity under the evaluated period and conditions (Figure 2).

**Figure 2:**
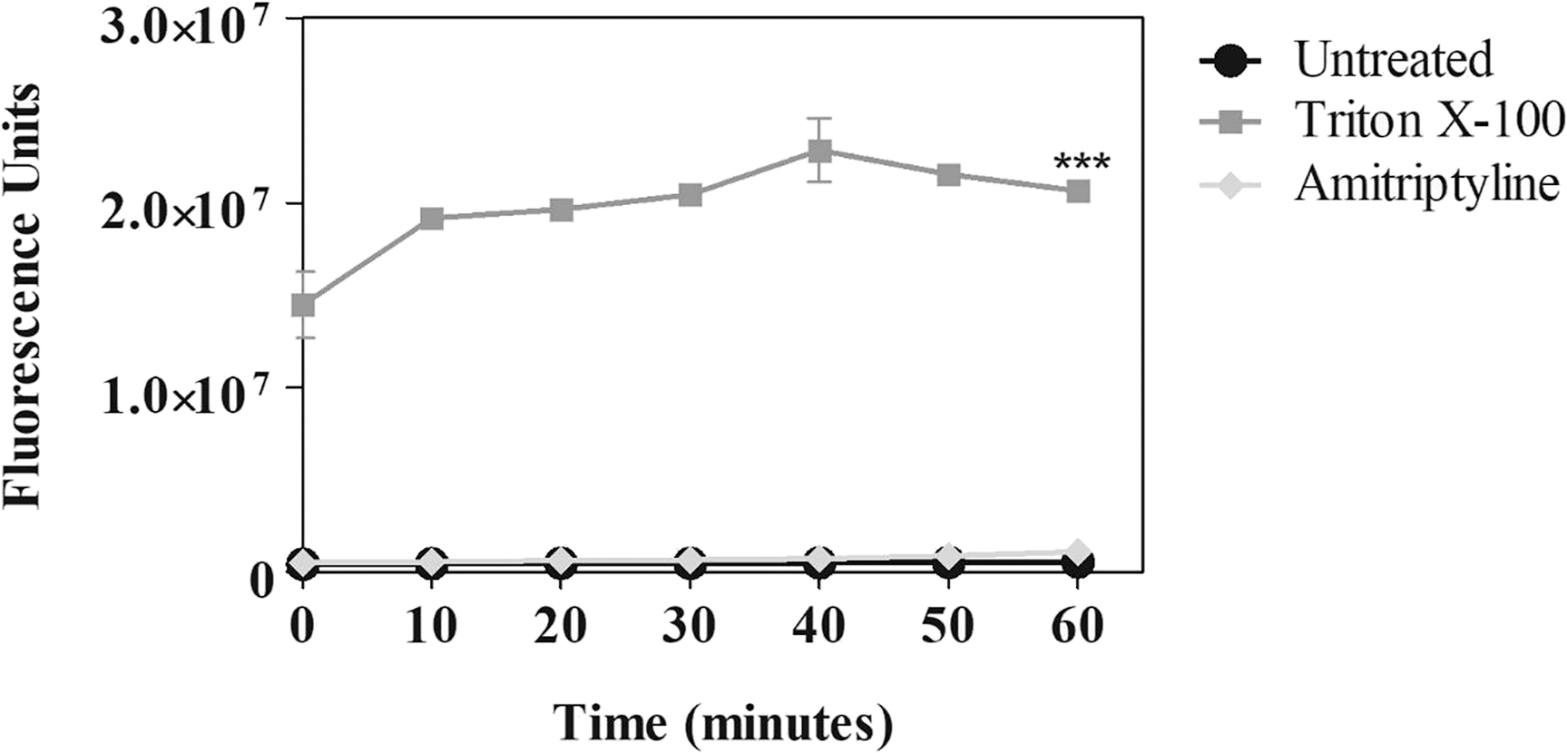
Evaluation of plasma membrane integrity in *Leishmania (Leishmania) amazonensis* promastigotes following amitriptyline treatment using SYTOX Green probe. Controls included untreated cells and cells treated with Triton X-100 (maximum permeabilization). The p-value is represented by *** (*p* < 0.0001). Bars represent the mean and standard deviation of duplicates from a representative assay.

To evaluate mitochondrial membrane potential in *L. (L.) amazonensis* promastigotes, the rhodamine 123 probe was used, and fluorescence was quantified by flow cytometry at 30 and 60 min. Promastigotes treated with amitriptyline at its EC_50_ concentration were assessed alongside untreated parasites and carbonyl cyanide p-trifluoromethoxyphenylhydrazone (FCCP) treated parasites. Unstained parasites served as an internal control for the experiment. Amitriptyline reduced fluorescence units by approximately 90% at both time-points compared to untreated parasites (***p < 0.0004), indicating mitochondrial membrane potential depolarization after 30 and 60 min of incubation. FCCP-treated parasites showed a significant reduction of approximately 85% relative to untreated parasites (***p < 0.0004) (Figure 3). This control was used to indicate mitochondrial membrane potential depolarization.

**Figure 3:**
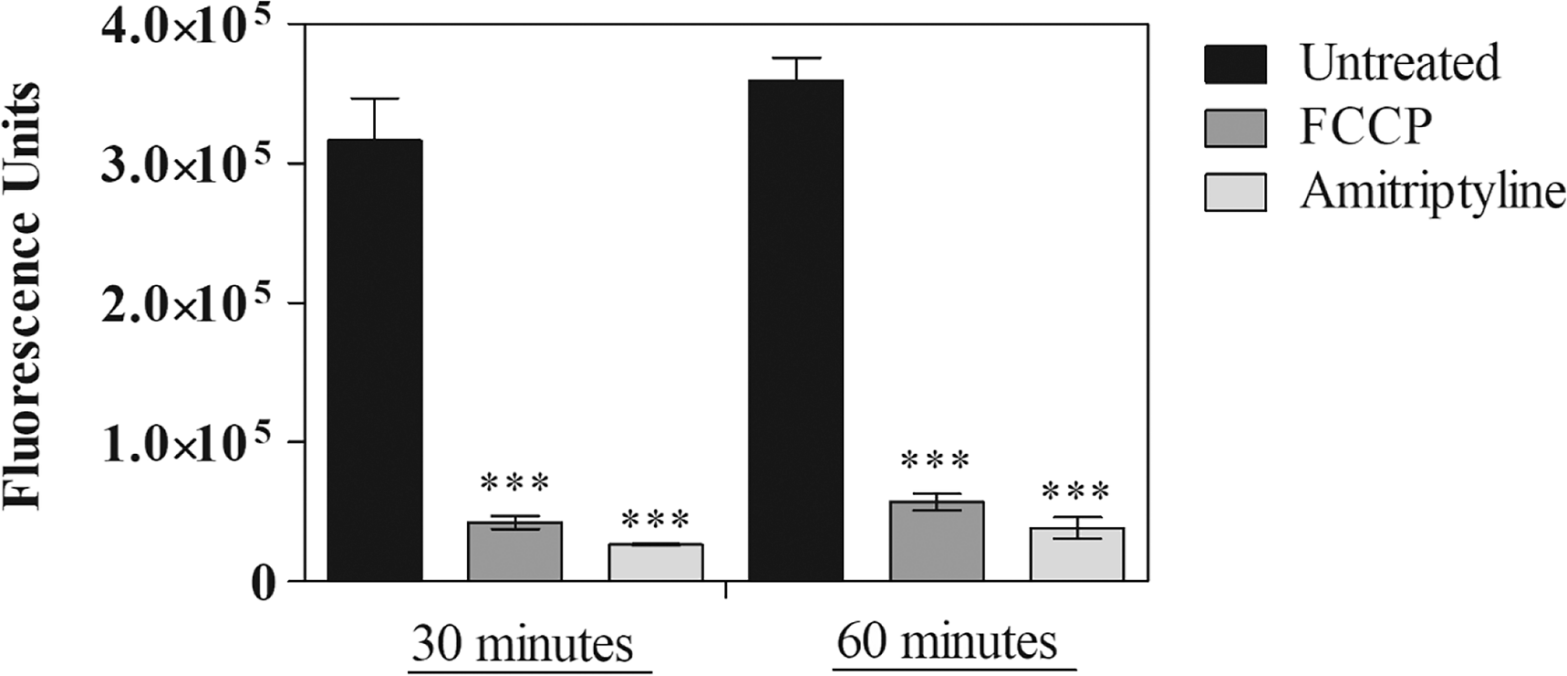
Evaluation of mitochondrial membrane potential using rhodamine 123 by flow cytometry. Promastigote forms were treated with amitriptyline at the drug’s EC_50_ for 30 and 60 min. Controls included untreated and FCCP-treated parasites. The graph represents a mean fluorescence unit and standard deviation of duplicates from a representative experiment. The p-value is represented by *** (*p* < 0.0004), indicating a significant difference from the 30 and 60 min untreated controls.

Fluorescence histograms representative of two independent experiments with amitriptyline and FCCP-treated parasites, together with unlabeled and untreated parasites are shown in Supplementary Figure 2.

To evaluate reactive oxygen species (ROS) production, the H_2_DCF-DA probe was used. Untreated parasites and parasites treated with sodium azide for 60 min were used as controls. Untreated parasites served as the baseline for ROS production, while sodium azide-treated parasites represented cells with increased ROS production, showing a significant increase relative to the control group (***p < 0.0001). After incubation with amitriptyline for 60 min, fluorescence units remained comparable to those of untreated cells, indicating no increase or decrease in ROS production (Figure 4).

**Figure 4:**
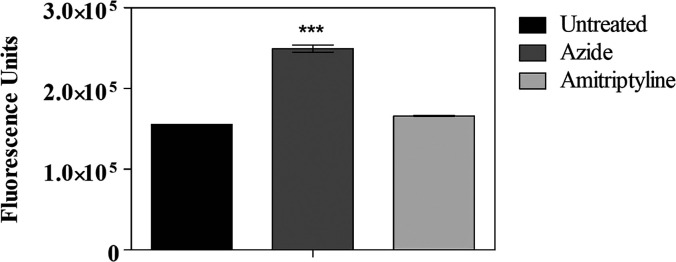
Evaluation of ROS production using the H_2_DCF-DA probe. Promastigote forms were treated with amitriptyline at EC_50_ for 60 min. Controls included untreated cells and sodium azide-treated cells. The *p*-value is represented by *** (*p* < 0.0001), indicating a statistical difference from the untreated control. Bars represent the mean and standard deviation of duplicates from a representative experiment.

To evaluate the ultrastructural changes induced by amitriptyline in parasites and to identify potential organelle targets of this drug, *L. (L.) amazonensis* promastigotes were treated with amitriptyline at its EC_5__0_ concentration for 15, 30, and 60 min. Untreated promastigotes were used as experimental controls (Figure 5A). These control parasites exhibited preserved nuclei (n), plasma membranes (pm), mitochondria (m), and kinetoplasts (k) (Figure 5A).

**Figure 5:**
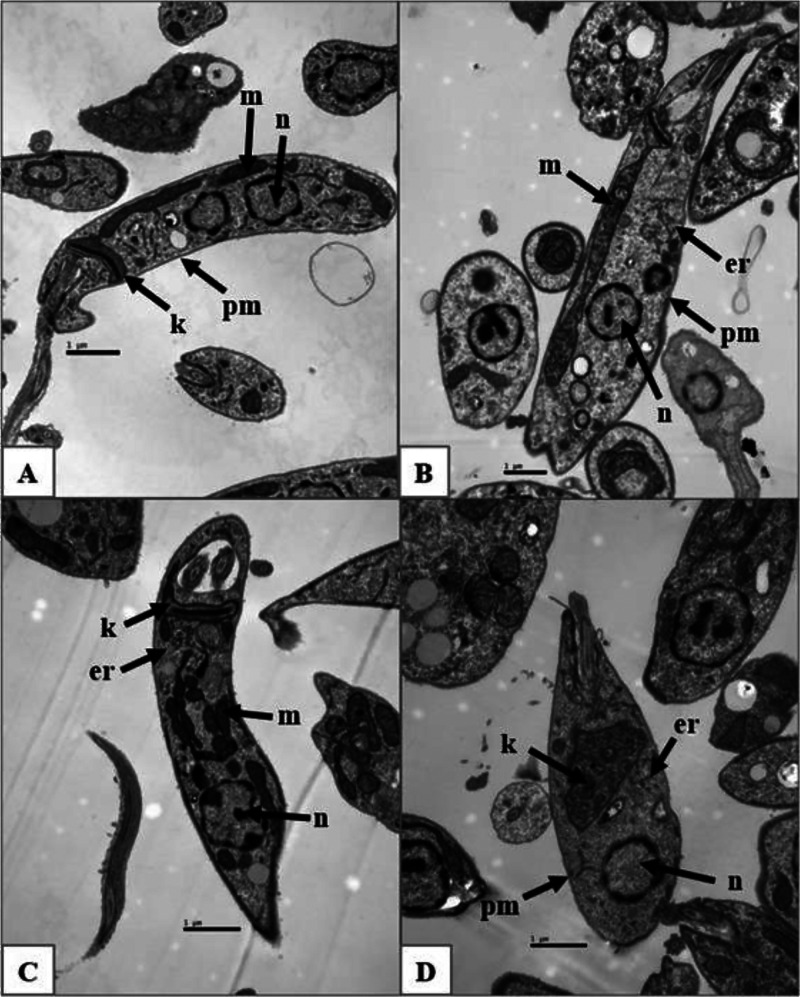
Ultrastructural analysis of promastigotes treated with amitriptyline at EC_50_ concentration for 15, 30, and 60 min. Images acquired by transmission electron microscopy. Untreated promastigotes (A: scale bar 1 μm) show preserved nuclei (n), plasma membranes (pm), mitochondria (m), and kinetoplasts (k). Promastigotes treated with amitriptyline for 15 min (B: scale bar 1 μm) exhibit mitochondria slightly swollen. Promastigotes treated for 30 min (C: scale bar 1 μm) show a range of variation in mitochondrial size and shape. Promastigotes treated for 60 min (D: scale bar 1 μm) present severe mitochondrial swelling. Fragmentation of the endoplasmic reticulum (er) was observed at all time points evaluated. Magnification 15,000×.

Figure 5B shows representative images of promastigotes treated with amitriptyline for 15 min, in which mitochondria appear slightly swollen (indicated by arrows), while the plasma membrane morphology remains similar to that of untreated cells. Figure 5C,D depict promastigotes treated for 30 and 60 min, respectively. Parasites treated for 30 min display marked variations in mitochondrial size and shape, as well as an unusual degree of pleomorphism. At 60 min, promastigotes show severe mitochondrial swelling. Fragmentation of the endoplasmic reticulum (er) was observed at all time points evaluated.

### 2.3 Therapeutic Combinations

Therapeutic combinations were also tested to investigate possible synergy or additivity between the antileishmanial activity of amitriptyline and the standard drugs amphotericin B and miltefosine. Promastigote and intracellular amastigote forms were treated using a modified fixed ratio isobologram method for 48 h. At the end of each combination experiment, dose–response curves were generated according to the different proportions in which the drugs were tested (5:0, 4:1, 3:2, 2:3, 1:4, and 0:5), resulting in EC_50_ values for each drug and the combinations (data not shown). These values were used to calculate the fractional inhibitory concentration (FIC), the sum of FIC (ΣFIC), the mean sum of FIC (XΣFIC) and to construct isobolograms.

As shown in Table 2, the XΣFIC values generated by the combination of amitriptyline with standard drugs amphotericin B and miltefosine in *L. (L.) amazonensis* promastigotes and intracellular amastigotes forms ranged from 1.0 to 1.8. According to the XΣFIC values, which represent the overall behavior of the combination, the interactions were classified as additive or indifferent, with values falling between 0.5 and 4.

**Table 2:** Mean Sum of Fractional Inhibitory Concentration (XΣFIC) Values Generated by the Combination of Amitriptyline with Standard Drugs Amphotericin B and Miltefosine in *Leishmania (Leishmania) amazonensis* Promastigotes and Intracellular Amastigotes Forms^a^

therapeutic combination	*Leishmania* forms	X∑FIC ± SD
amitriptyline + amphotericin B	promastigotes	1.2 ± 0.2
amitriptyline + miltefosine	promastigotes	1.0 ± 0.1
amitriptyline + amphotericin B	amastigotes	1.8 ± 0.1
amitriptyline + miltefosine	amastigotes	1.3 ± 0.3

a X∑FIC: mean sum of fractional inhibitory concentration. The results are expressed as the mean and standard deviation (SD) of two independent experiments, each one performed in duplicate.

Isobolograms resulting from the combination of amitriptyline with the standard drugs are shown in Figure 6. Values less than or equal to 0.5 were considered synergistic, values between 0.5 and 4 were considered additive or indifferent, and values greater than 4 were considered antagonistic. Under this conservative classification, the combinations of amitriptyline with amphotericin B or miltefosine resulted in additive or indifferent interactions.

**Figure 6:**
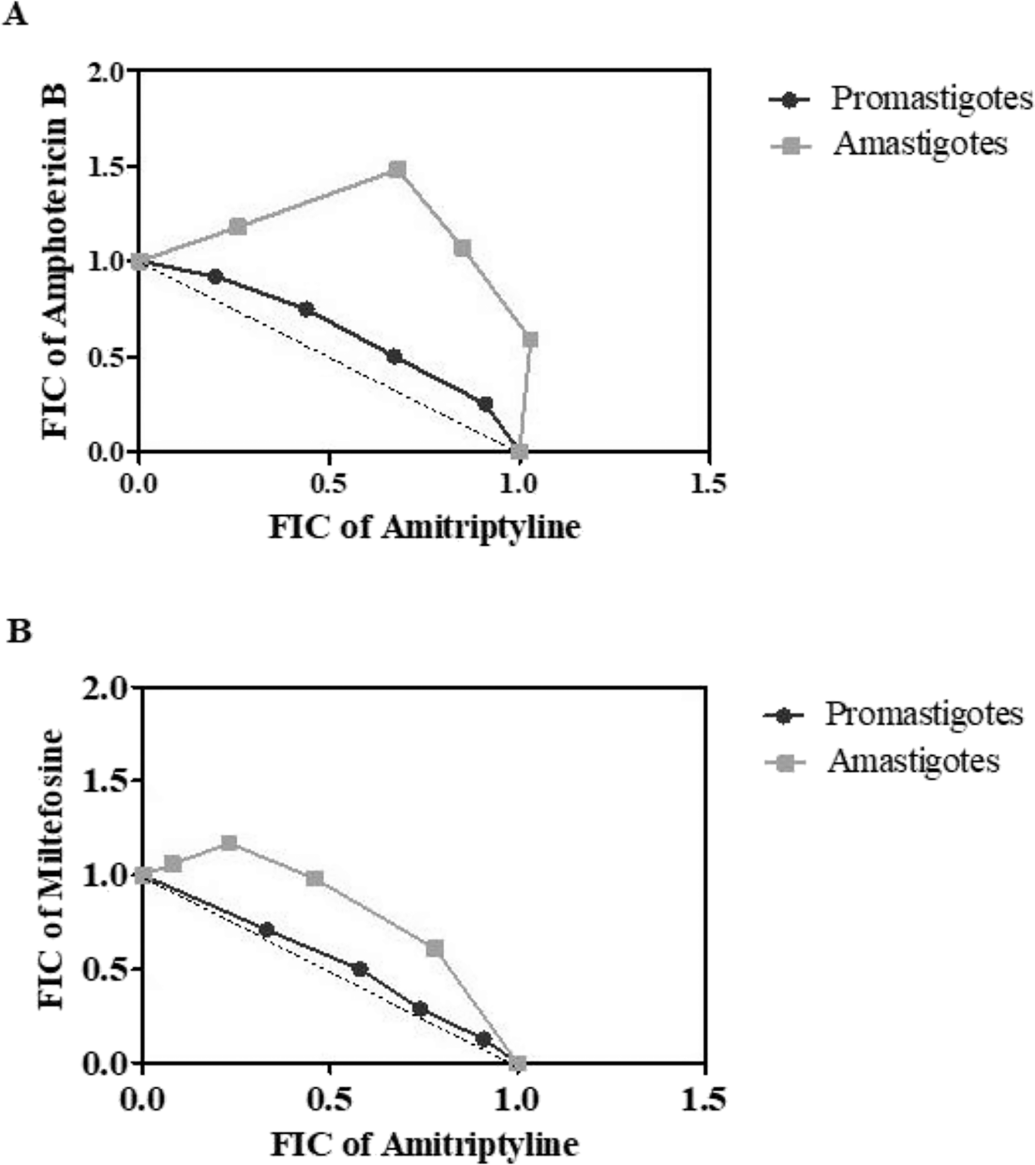
Isobologram constructed based on the Fractional Inhibitory Concentration (FIC) values from the combination of amitriptyline and amphotericin B (A) or miltefosine (B) in promastigotes and intracellular amastigotes of *Leishmania (Leishmania) amazonensis*. The dotted line corresponds to the predicted position of the experimental points for a simple additive effect. Values represent an average of two independent experiments.

## 3 Discussion

As evidenced by our findings, the strategy of repurposing amitriptyline for leishmaniasis holds promise due to its antiparasitic effects and additive interactions with the clinically used drugs amphotericin B and miltefosine. Amitriptyline, a tricyclic antidepressant (TCA), belongs to a class of compounds with established clinical applications for treating mood disorders, neuropathic pain, and migraines.^13^ Other TCAs, such as nortriptyline and imipramine, share similar pharmacological profiles and therapeutic uses. Given the extensive clinical use and documented safety of TCAs, exploring amitriptyline for leishmaniasis could significantly expedite the availability of an effective, alternative therapeutic option for this parasitic neglected disease.

This study provides insights into the antileishmanial activity of amitriptyline against *L. (L.) amazonensis* promastigote and intracellular amastigote forms. The compound demonstrated selective antileishmanial activity, with a lethal effect at an unharmful concentration to mammalian host cells, indicating a potential therapeutic value. Furthermore, the absence of increased SYTOX Green probe uptake indicates that amitriptyline does not compromise the plasma membrane integrity of promastigotes at initial incubation, a finding that points to alternative cellular targets underlying its leishmanicidal action.

The luminescence-based assay was used to determine parasite burden in infected macrophages. This method is widely accepted for quantifying intracellular parasites and is recognized for its superior sensitivity and reproducibility compared to manual counting of Giemsa-stained slides.^15^

Cytotoxicity was assessed using the NCTC clone 929 fibroblast cell line, a validated model for early stage screening.^16^ Although BMDMs were used in the intracellular efficacy assay, cytotoxicity in primary macrophages was not performed due to ethical constraints. While we acknowledge this as a limitation, the choice aligns with the 3Rs principle (Replacement, Reduction and Refinement) and is justified by the repurposing nature of amitriptyline, a clinically approved drug with a well-established toxicological profile in humans.

The higher CC_5__0_ values observed for miltefosine and amphotericin B in NCTC cells, when compared to primary macrophages, are consistent with previously reported data and reflect intrinsic differences in cellular susceptibility. Fibroblasts, such as the NCTC-929 cell line, generally exhibit lower sensitivity to cytotoxic agents due to distinct metabolic and structural characteristics.^16^ For example, miltefosine CC_5__0_ values have been reported at 49 μM in primary macrophages^17^ versus 116–241 μM in fibroblasts,^18–20^ aligning with the values obtained in our study. Although primary phagocytic cells provide higher physiological relevance for infection models, fibroblast-based assays remain a valid and widely used tool for early stage cytotoxicity screening.

The investigation of mitochondrial membrane potential revealed significant mitochondrial depolarization in promastigotes treated with amitriptyline, which was comparable to the effect observed with the positive control FCCP, a known mitochondrial uncoupler. Mitochondrial depolarization is particularly detrimental to *Leishmania* parasites due to their reliance on aerobic metabolism for ATP production. Disruption of mitochondrial function impairs energy homeostasis, leading to parasite death. This mechanism aligns with the observed ultrastructural damage to mitochondria, further supporting the hypothesis that amitriptyline targets essential metabolic processes in *Leishmania*. This suggests that amitriptyline induces mitochondrial dysfunction as part of its antileishmanial mechanism. Given the essential role of mitochondria in parasite survival and energy metabolism, amitriptyline induced mitochondrial depolarization appears to be a key contributor to parasite death. However, no significant change in ROS levels was observed following amitriptyline treatment, suggesting that its leishmanicidal effect is not mediated by oxidative stress. These findings are consistent with previous studies that identify mitochondrial collapse as a key mechanism of antileishmanial activity in various FDA-approved drugs. For example, fendiline treatment in *L. (L.) infantum* led to mitochondrial membrane depolarization without disrupting the plasma membrane^21^; nitazoxanide rapidly altered promastigote metabolism by inducing mitochondrial membrane depolarization^22^; and the antidepressant sertraline caused respiratory uncoupling, a significant reduction in intracellular ATP levels, and oxidative stress in *L. (L.) infantum* promastigotes.^23^ Although ATP quantification was not performed in this study, the extent of mitochondrial dysfunction strongly suggests impaired bioenergetics and possible ATP depletion, as also reported for related tricyclic antidepressants as sertraline^23^ and cyclobenzaprine.^24^ Amitriptyline is a tricyclic compound chemically related to cyclobenzaprine; both share central nervous system activity and exhibit similar histaminergic, anticholinergic, and noradrenergic receptor binding profiles. In our previous studies, cyclobenzaprine was shown to decrease intracellular ATP levels in *Leishmania*, leading to irreversible depolarization of the plasma membrane.^18^ We propose the inclusion of ATP assays in future studies to confirm this hypothesis.

Electron microscopy observations provided additional evidence of the impact of amitriptyline on parasite ultrastructure. Treated promastigotes exhibited marked mitochondrial imbalance, with noticeable variations in mitochondrial size and shape, as well as an unusual degree of pleomorphism after 30 min of exposure—features that may compromise mitochondrial integrity and function. After 60 min, severe mitochondrial swelling was observed. In addition, fragmentation of the endoplasmic reticulum was noted, which could interfere with the parasite’s protein synthesis. This structural damage, observed in the absence of plasma membrane disruption, supports the hypothesis that amitriptyline specifically targets intracellular organelles rather than inducing cell lysis through membrane destabilization.

Our findings on the antileishmanial activity of amitriptyline aligning with existing literature, highlighting several mechanisms through which tricyclic compounds, including amitriptyline, may impair *Leishmania* spp. cellular functions. In the study by Zilberstein and co-workers (1990), tricyclic drugs such as imipramine were observed to reduce the proton motive force of *L. (L.) donovani* promastigotes, specifically through the inhibition of proline transport, ATP depletion, and membrane potential reduction.^9^ Our results confirm a similar disruption of cellular homeostasis, where amitriptyline affects mitochondrial and endoplasmic reticulum, supporting that its effect may involve an energetic imbalance. Study by Lima and co-workers (2022) with cyclobenzaprine, a structurally related drug, also emphasized a mitochondrial disruption and ATP level reduction in *Leishmania*, a similar effect observed in amitriptyline treated parasites.^24^ Tonelli and co-workers (2020) have previously demonstrated amitriptyline analogues with potent antileishmanial activity and low cytotoxicity in host cells, indicating the therapeutic potential of this drug.^25^ The existing studies underline the advantage of repurposing tricyclics like amitriptyline for antileishmanial therapy, suggesting that mechanisms such as mitochondrial dysfunction and cellular metabolism targeting are promising strategies in developing effective antileishmanial treatments.

When combined with standard antileishmanial drugs, amphotericin B and miltefosine, amitriptyline exhibited additive or indifferent interactions, as indicated by the FIC values and isobolographic analyses. The absence of synergism may be attributed to the distinct mechanisms of action of amitriptyline and the reference drugs, which do not potentiate each other’s effects. Nonetheless, the additive effects suggest that amitriptyline could still be valuable in combination therapy, potentially allowing dose reductions of amphotericin B, a drug with several adverse effects. Additionally, the metabolization of both drugs significantly differs, while the metabolism of amphotericin B is exclusively renal,^26^ the metabolism of amitriptyline occurs mainly by demethylation (CYP2C19, CYP3A4) as well as hydroxylation (CYP2D6) followed by conjugation with glucuronic acid,^14^ offering opportunities to combining both drugs.

Although no synergism was detected, the additive behavior observed in several combinations suggests that amitriptyline could serve as an adjunct therapy, potentially allowing dose reductions of these conventional drugs and possibly minimizing their associated toxicity. The lack of antagonism further highlights the amitriptyline compatibility with established antileishmanial treatments, which could support its inclusion in combination therapy strategies where drug tolerance and resistance are growing concerns. In addition to amphotericin B and miltefosine, amitriptyline could be explored in combination with other antileishmanial drugs, such as paromomycin or pentavalent antimonials. These combinations may offer further benefits, including enhanced efficacy, reduced treatment duration, and lower risk of resistance development. Given the additive interactions observed in this study, amitriptyline could serve as a versatile adjunct in multidrug regimens, potentially improving outcomes for patients with leishmaniasis. However, additional experiments are needed to confirm this possibility.

Besides, cutaneous leishmaniasis caused by *L. (L.) amazonensis* is a harmful disease and has the potential to spread to mucocutaneous areas, causing a disfiguring disease, impacting the life of patients. Orally or even topically administrations of amitriptyline may be in future a novel adjunctive therapeutic for cutaneous leishmaniasis, potentially exerting leishmanicidal effects that contribute to decreased parasite load.

## 4 Conclusions

This study demonstrates that amitriptyline exhibits promising in vitro antileishmanial activity against *L. (L.) amazonensis*, affecting both promastigote and intracellular amastigote forms. Amitriptyline induces structural alterations, particularly mitochondrial disruption, suggesting that its mechanism may involve interference with the essential cellular processes of energy metabolism, and strengthening its candidacy for drug repurposing in leishmaniasis treatment. Comparisons with other tricyclic compounds support the hypothesis that mitochondrial destabilization is a key mechanism of this drug class in *Leishmania* spp. Although further in vivo studies are necessary, our results indicate that amitriptyline holds significant promise as a drug candidate for leishmaniasis, potentially offering a cost-effective, accessible alternative to current treatments. This work contributes to the growing body of research supporting drug repositioning as a viable strategy for addressing the urgent need for new antileishmanial therapies.

## 5 Material and Methods

### 5.1 Materials

RPMI 1640 medium, M-199 medium, MTT, FCCP, amitriptyline, miltefosine, dimethyl sulfoxide (DMSO), methanol, and sodium dodecyl sulfate (SDS) were purchased from Sigma-Aldrich (Brazil). Fetal bovine serum (FBS) was obtained from Gibco. Hygromycin B, SYTOX Green, rhodamine 123, and H_2_DCF-DA were acquired from Molecular Probes (Invitrogen, Brazil). One-Glo Luciferase Assay System was purchased from Promega (Brazil). Amphotericin B was obtained from Cristália (Brazil).

### 5.2 Parasites and Mammalian Cell Maintenance

*Leishmania (Leishmania) amazonensis* wild-type (La-WT) (MHOM/BR/1973/M2269) promastigotes were cultured in M-199 medium supplemented with 10% heat-inactivated FBS and 0.25% hemin at 25 °C. The transgenic *Leishmania (Leishmania) amazonensis* line expressing luciferase (La-Luc) was maintained under the same conditions, with the addition of 32 μg/mL hygromycin B.^20^ Parasites were used up to passage 10. The strains were kindly provided by Professor Dr. Silvia Reni Bortolin Uliana (University of São Paulo, Brazil).

The NCTC clone 929 [L cell, L-929, derivative of Strain L] (mouse connective tissue) was cultured in RPMI 1640 medium supplemented with 10% heat-inactivated FBS and maintained in a humidified incubator at 37 °C with 5% CO_2_. Cells were passed twice a week and used up to passage 20.

Bone marrow-derived macrophages (BMDMs) were obtained from BALB/c mice, following previously described protocols.^15,27^ Female BALB/c mice (6 weeks old) were obtained from the animal breeding facility of the Adolfo Lutz Institute (São Paulo, Brazil). The animals were housed in sterilized cages under controlled environmental conditions, with free access to food and water (ad libitum). All animal experiments were approved by the Ethical Committee for Animal Experiments of the Adolfo Lutz Institute, São Paulo State Health Department (CEUA 04/2016, Brazil).

### 5.3 Antileishmanial Activity and Cytotoxicity against Mammalian Cells

To evaluate the antileishmanial activity and cytotoxicity against mammalian cells, the EC_50_ and the CC_50_ of each tested drug were determined. The drugs used in this study were prepared as follows: amitriptyline was solubilized in DMSO, while amphotericin B and miltefosine were solubilized in sterile distilled water.

#### 5.3.1 Activity against Promastigotes

Late-growth phase promastigotes (La-WT) were washed with medium and seeded at a density of 1 × 10^6^/well. The drugs were diluted in M-199 medium in 96-well microplates, with a maximum concentration of 200 μM for amitriptyline and miltefosine, and 1 μM for amphotericin B. Serial 2-fold dilutions of the drugs were prepared directly in the microplates. Amphotericin B and miltefosine were used as reference drugs. Untreated and drug-treated promastigotes were incubated for 48 h at 25 °C, and their viability was assessed by mitochondrial activity using the MTT assay.^28^ Optical density was measured at 570 nm using a plate reader (FilterMax F5Multi-Mode Microplate Reader). Data represents the mean and standard deviation of two independent experiments.

#### 5.3.2 Activity against Intracellular Amastigotes

Bone marrow-derived macrophages (BMDMs) were obtained from BALB/c mice and differentiated as previously described.^27^ Macrophages were seeded in 96-well microplates at a density of 1 × 10^5^/well in RPMI medium supplemented with 10% FBS and incubated for 24 h at 37 °C in a humidified incubator with 5% CO_2_.

Macrophages were then infected with stationary-phase promastigotes (La-Luc) at a parasite-to-macrophage ratio of 20:1 for 4 h at 33 °C in a 5% CO_2_ humidified incubator.^15^ After incubation, noninternalized parasites were removed, and fresh RPMI medium supplemented with 10% FBS and the tested drugs was added. The maximum concentrations used were 150 μM for amitriptyline, 0.4 μM for amphotericin B, and 40 μM for miltefosine. The cells were incubated for 48 h.

To assess macrophage infection, the parasite load was quantified using the One-Glo luciferase assay in 96-well microplates, as described.^15^ Luminescence units were measured using a microplate reader (POLARstar Omega, BMG Labtech, Ortenberg, Germany), with the luminescence reading of uninfected macrophages used as a baseline. Untreated infected macrophages and infected macrophages treated with standard drugs were used as experimental controls. Data represents the mean and standard deviation of two independent experiments.

#### 5.3.3 Cytotoxicity against Mammalian Cells

NCTC clone 929 cells were seeded at a density of 6 × 10^4^ cells/well in 96-well microplates and exposed to a maximum concentration of 300 μM for amitriptyline and 400 μM for amphotericin B and miltefosine. Untreated and drug-treated cells were incubated for 48 h at 37 °C in a humidified incubator with 5% CO_2_ to determine the CC_50_ value.

Cell viability was assessed using the MTT assay,^29^ and optical density was measured at 570 nm using a plate reader (FilterMax F5Multi-Mode Microplate Reader). The selectivity index (SI) was calculated using the ratio CC_50_ (against mammalian cells)/EC_50_ (against intracellular parasites).^20^ Data represents the mean and standard deviation of two independent experiments.

#### 5.3.4 Therapeutic Combinations

The interactions between amitriptyline and amphotericin B or miltefosine were determined using the modified isobologram method.^30,31^ First, the EC_50_ values of each drug were determined individually. These assays have been described previously (Sections 5.3.1 and 5.3.2).

After determining the EC_50_ values for each drug, the maximum concentrations of individual drugs were calculated to ensure that the EC_50_ was positioned at the midpoint of the serial dilution microplate.^30^ The highest concentrations of drug solutions were prepared in proportions of 5:0, 4:1, 3:2, 2:3, 1:4, and 0:5 for drug A and drug B, respectively, and then serially diluted 2-fold across the microplate.

To determine the nature of the interaction and construct the isobologram, the FIC index was calculated as the ratio between the EC_50_ values of the combined drugs and the EC_50_ values of each drug alone. The ∑FIC and the X∑FIC were calculated. Isobolograms were constructed by plotting the FIC for each drug ratio. The X∑FIC was used to classify the interaction as recommended by Odds (2003), with interactions categorized as synergistic (X∑FIC ≤ 0.5), additive or indifferent (X∑FIC > 0.5 and ≤ 4) or antagonistic (X∑FIC > 4).^32^

### 5.4 Mechanistic Studies

#### 5.4.1 Plasma Membrane Integrity

Late-growth phase promastigotes were washed with PBS, seeded at a density of 2 × 10^6^/well, and incubated with 1 μM SYTOX Green in a black 96-well microplate for 15 min in the dark at 25 °C.^22,33^ Amitriptyline was added at its EC_50_ concentration, and two controls were used: (a) maximum permeabilization (0.1% Triton X-100) and (b) untreated promastigotes (normal plasma membrane).

Fluorescence was measured every 10 min for up to 60 min using a fluorometric microplate reader (FilterMax F5Multi-Mode Microplate Reader, Molecular Devices) with excitation and emission wavelengths of 485 and 520 nm, respectively. Internal controls included (a) detection of possible amitriptyline fluorescence at the appropriate wavelengths and (b) detection of possible interference from DMSO. Data represents the mean and standard deviation of two independent experiments.

#### 5.4.2 Mitochondrial Membrane Potential

Late-growth phase promastigotes were washed with PBS, seeded at a density of 2 × 10^6^/well, and incubated with amitriptyline at its respective EC_50_ value for 30 and 60 min at 25 °C. Rhodamine 123 (0.3 μg/mL) was then added, and the promastigotes were incubated for 10 min in the dark at 37 °C.^19^

After incubation, cells were washed with PBS and analyzed by flow cytometry (Attune NxT Acoustic Focusing Cytometer, Thermo Fisher Scientific) using a 488 nm excitation filter, with 10,000 events recorded per sample using the Attune NxT software. FCCP (20 μM) was used as a positive control for mitochondrial membrane potential depolarization, while untreated cells were used as a control for normal mitochondrial membrane potential.^19,34^ Data represents the mean and standard deviation of two independent experiments.

#### 5.4.3 Reactive Oxygen Species (ROS)

Late-growth phase promastigotes were washed with PBS, seeded at a density of 2 × 10^6^/well, and incubated with amitriptyline at its respective EC_50_ value in a black 96-well microplate for 60 min at 25 °C. H_2_DCFDA (5 μM) was then added, and the promastigotes were incubated for an additional 15 min in the dark at 25 °C.^22^

Fluorescence intensity was measured using a fluorometric microplate reader (FilterMax F5Multi-Mode Microplate Reader, Molecular Devices) with excitation and emission wavelengths of 485 and 520 nm, respectively. Sodium azide (10 mM) was used as a positive control (inducing increased ROS production), while untreated promastigotes served as a control for normal ROS production.^35^ Internal controls were used as described above. Data represents the mean and standard deviation of two independent experiments.

#### 5.4.4 Ultrastructural Evaluation by Transmission Electron Microscopy

Late-growth phase promastigotes were washed with PBS, seeded at a density of 2 × 10^7^/well, and incubated with amitriptyline at its respective EC_50_ value for 15, 30, and 60 min at 25 °C. Untreated promastigotes were used as a control group (showing the normal ultrastructure of the promastigotes).

After the incubation period, the promastigotes were washed, fixed in 2.5% glutaraldehyde, postfixed in 1% osmium tetroxide, dehydrated in an acetone series, and embedded in Epon 812 resin, as previously described.^36^ The samples were analyzed using a Transmission Electron Microscope (JEOL).

### 5.5 Statistical Analysis

The EC_50_ and CC_50_ values were determined using sigmoidal dose–response curves generated in GraphPad Prism 5.0 software. Differences between samples and controls were statistically evaluated using one-way ANOVA followed by Tukey’s multiple comparison test to determine the p-value. A p-value of <0.05 was considered statistically significant.

## Supplementary Material



## Notes

Representative dose–response curves of amitriptyline, amphotericin B, and miltefosine against *Leishmania (L.) amazonensis* promastigotes (A), intracellular amastigotes (B), and NCTC clone 929 cells (C), representative curves from two independent experiments; representative fluorescence histograms from two independent experiments showing *Leishmania (L.) amazonensis* promastigotes labeled with Rhodamine 123 after treatment with amitriptyline or FCCP, compared to untreated and unlabeled controls, at 30 min (A) and 60 min (B). Results demonstrate a reduction in mitochondrial membrane potential following amitriptyline treatment. (PDF)
