# Supplementary material for: Mechanistic Insights and Therapeutic Potential of the Antidepressant Amitriptyline against Leishmania (Leishmania) amazonensis
Source: ACS Omega. 2025 Aug 7;10(32):36432–40. doi: 10.1021/acsomega.5c04856 (PMC12368814; doi:10.1021/acsomega.5c04856)
Supplement: Supplementary file 1 [file ao5c04856_si_001.pdf]

Mechanistic Insights and Therapeutic Potential of the Antidepressant Amitriptyline Against *Leishmania (Leishmania) amazonensis*

Juliana Tonini Mesquita[1], Noemi Nosomi Taniwaki[2], Andre Gustavo Tempone[1*], Juliana Quero Reimão[3*]

[1]Instituto Butantan, Pathophysiology Laboratory, São Paulo, SP, Brazil.

[2] Instituto Adolfo Lutz, Electron Microscopy Nucleus, São Paulo, SP, Brazil.

[3] Faculdade de Medicina de Jundiaí, Laboratory of Preclinical Assays and Research of Alternative Sources of Innovative Therapy for Toxoplasmosis and Other Sicknesses (PARASITTOS), Jundiaí, SP, Brazil.

*corresponding authors:

Andre Gustavo Tempone: andre.tempone@butantan.gov.br

Juliana Quero Reimão: julianareimao@g.fmj.br

Supporting Information

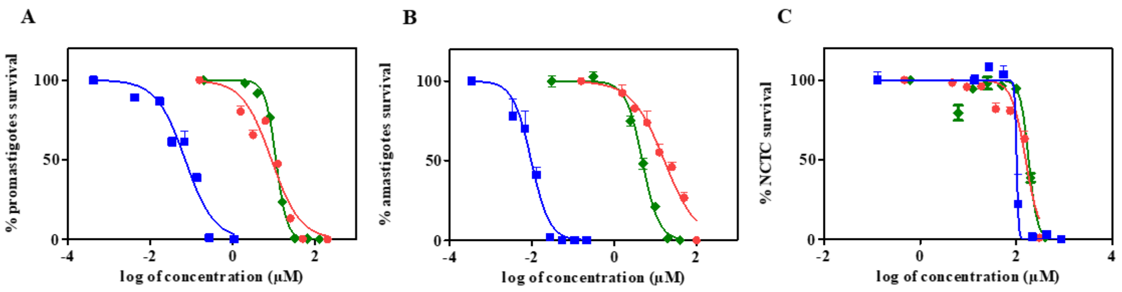

Supplementary Figure 1. Representative dose–response curves of amitriptyline, amphotericin B, and miltefosine against *Leishmania* (L.) *amazonensis* promastigotes (A), intracellular amastigotes (B), and NCTC clone 929 cells (C). Representative curves from two independent experiments. ● Amitriptyline, ■ Amphotericin B, ◆ Miltefosine.

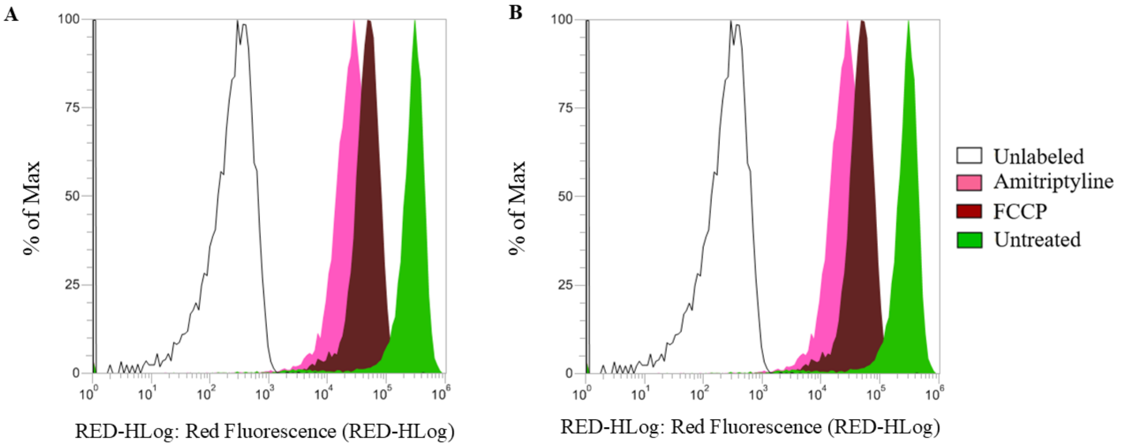

Supplementary Figure 2. Representative fluorescence histograms from two independent experiments showing *Leishmania* (L.) *amazonensis* promastigotes labeled with Rhodamine 123 after treatment with amitriptyline or FCCP, compared to untreated and unlabeled controls, at 30 minutes (A) and 60 minutes (B). Results demonstrate a reduction in mitochondrial membrane potential following amitriptyline treatment.
